# MAPK/ERK Signaling Pathway in Hepatocellular Carcinoma

**DOI:** 10.3390/cancers13123026

**Published:** 2021-06-17

**Authors:** Hyuk Moon, Simon Weonsang Ro

**Affiliations:** Department of Genetics and Biotechnology, College of Life Sciences, Kyung Hee University, Yongin-si 17104, Gyeonggi-do, Korea; HMOON@khu.ac.kr

**Keywords:** hepatocellular carcinoma, MAPK/ERK signaling, animal models, targeted therapies

## Abstract

**Simple Summary:**

The mitogen-activated protein kinase/extracellular signal-regulated kinase (MAPK/ERK) signaling pathway is frequently activated in liver cancer, which is one of the most lethal cancers in humans. In addition to genetic mutation leading to persistent activation of effector molecules in the MAPK/ERK signaling cascade, there are alternative means by which the MAPK/ERK signaling pathway is activated in cancer. In this review, we will introduce the diverse modulators regulating the MAPK/ERK signaling pathway and consider the possibility of targeting the effectors and regulators in order to suppress the pro-tumorigenic MAPK/ERK signaling pathway, especially in liver cancer.

**Abstract:**

Hepatocellular carcinoma (HCC) is a major health concern worldwide, and its incidence is increasing steadily. Recently, the MAPK/ERK signaling pathway in HCC has gained renewed attention from basic and clinical researchers. The MAPK/ERK signaling pathway is activated in more than 50% of human HCC cases; however, activating mutations in RAS and RAF genes are rarely found in HCC, which are major genetic events leading to the activation of the MAPK/ERK signaling pathway in other cancers. This suggests that there is an alternative mechanism behind the activation of the signaling pathway in HCC. Here, we will review recent advances in understanding the cellular and molecular mechanisms involved in the activation of the MAPK/ERK signaling pathway and discuss potential therapeutic strategies targeting the signaling pathway in the context of HCC.

## 1. Introduction

The World Health Organization reported that the number of deaths due to liver cancer reached 830,000 in 2020 and ranked it the third most lethal cancer. Around one million people were diagnosed with HCC in 2020 [1,2]. Hepatocellular carcinoma accounts for up to 80% of all primary liver cancers, and its incidence is expected to continuously rise, representing a major global health problem [2].

Epidemiological and molecular studies have demonstrated that the development of HCC spans several decades. Patients with hepatitis B (HBV) or hepatitis C (HCV) chronic infection, especially when accompanied by liver cirrhosis, are at a much higher risk of developing HCC than healthy people. Other risk factors for HCC include alcohol abuse, diabetes, obesity, and metabolic syndromes [3]. The chronic inflammation caused by all these risk factors promotes hepatic fibrosis, which leads to hepatic cirrhosis and eventually to HCC [2].

HCC is a phenotypically and genetically heterogeneous tumor and its tumorigenesis is driven by a variety of molecular mechanisms [4]. Recent advances in molecular pathogenesis studies have defined various molecular signaling pathways that are critical to tumor initiation, progression, and metastasis in HCC. They include the mitogen-activated protein kinase/extracellular signal-regulated kinase (MAPK/ERK), phosphatidylinositol 3-kinase /AKT/mammalian target of rapamycin (PI3K/AKT/mTOR), Wnt/β-catenin, Janus kinase/signal transducer activator of transcription factor (JAK/STAT), Hedgehog (HH), and the Hippo signaling pathways. As the knowledge on oncogenic molecular pathways in HCC accumulates, there is growing interest in investigating novel therapeutic targets for key signaling molecules [4]. Of the signaling pathways identified in relation to HCC, the MAPK/ERK signaling pathway is known to be the most crucial pathway in HCC development [5].

In this review, we describe the cellular and molecular mechanisms involved in the activation of the MAPK/ERK signaling pathway and clinical trials targeting the signaling pathway in HCC. In addition, we propose new, promising therapeutic options targeting the MAPK/ERK signaling pathway. 

## 2. MAPK/ERK Signaling Pathway in HCC

### 2.1. The MAPK/ERK Signaling Pathway

There are at least three different MAPK signaling pathways that transduce extracellular signals into the nucleus to induce responsive genes in mammalian cells, including ERK, c-Jun NH2-terminal kinase (JNK), and p38. The ERK kinase family consists of ERK1 (p44) and ERK2 (p42). The JNK kinase family consists of JNK1, JNK2, and JNK3. Finally, the p38 MAPK family is divided into the following groups: p38α, p38β, p38γ, and p38δ [6]. Signals majorly initiated by growth factors are closely related to the ERK signaling pathway, and JNK and p38 signaling are activated by several factors, such as cytokines, growth factors, environmental stresses, and other stimuli [6]. This review will focus on the MAPK/ERK signaling pathway with particular attention paid to modulators of this signaling in HCC.

The MAPK/ERK signaling pathway is activated through signal transduction from cell surface receptors such as receptor tyrosine kinases (RTKs) or G-protein-coupled receptors (GPCRs) [7]. Improper regulation of the pathway leads to abnormal cellular behavior, including increased cell growth and proliferation, de-differentiation, and survival, which all promote carcinogenesis [7]. Receptors that can activate the MAPK/ERK signaling pathway include the epidermal growth factor receptor (EGFR), the fibroblast growth factor receptor (FGFR), the platelet-derived growth factor receptor (PDGFR), the vascular endothelial growth factor receptor (VEGFR), the insulin-like growth factor receptor (IGFR), the hepatocyte growth factor receptor (HGFR; also known as c-Met), and the stem cell growth factor receptor (SCFR; also known as KIT) [4].

**Figure 1:**
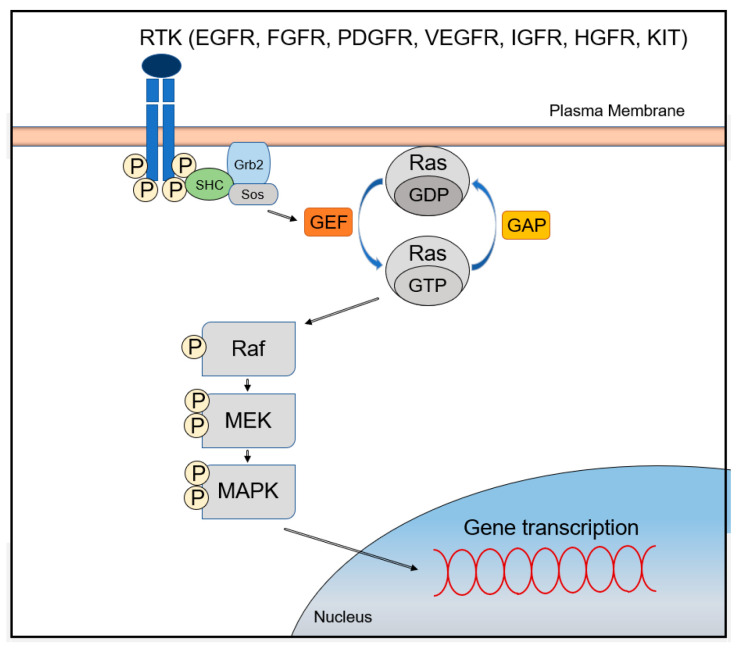
The MAPK/ERK signaling pathway.

Ligand binding to these receptors leads to the activation of cytoplasmic tyrosine kinases (TKs), which phosphorylate tyrosine residues at the cytoplasmic tails. This event recruits the growth-factor-receptor bound-2 (GRB2)/Shc/son of sevenless (SOS) adapter molecular complexes to the plasma membrane, which subsequently convert GDP-bound RAS to active GTP-bound RAS. After RAS activation, serine/threonine kinase RAF (A-RAF, B-RAF, and C-RAF) is recruited to the cell membrane and activated in a complex series of processes including phosphorylation and dimerization with scaffolding complexes [8]. RAF proteins directly regulate mitogen/extracellular protein kinases (MEK1 and MEK2), which ultimately leads to phosphorylation of the downstream signaling extracellular signal-regulated kinases (ERK1 and ERK2; also known as MAPK3 and MAPK1) [4]. Interestingly, MEKs are tyrosine and serine/threonine dual-specificity kinases [4]. Phosphorylated ERKs translocate into the nucleus, activating two key transcription factors of the AP-1 family, c-JUN and c-FOS [8]. These two factors induce the transcription of genes involved in cell cycle progression and cellular processes by binding to the AP-1 binding site of the promoter regions (Figure 1) [7]. Furthermore, genes encoding growth factors have binding sites in their promoter regions for transcriptional activators, which are activated by the MAPK/ERK signaling pathway. Thus, abnormal activation of MAPK/ERK signaling pathway leads to self-sufficiency of proliferative signals and continuous stimulation of cell growth through the establishment of an autocrine/paracrine loop [9].

Since the MAPK/ERK signaling pathway plays a fundamental role in the control of key cellular processes, including cell survival and proliferation, its aberrant activation is associated with cellular transformation and carcinogenesis [9]. The Ets transcription factor, which is phosphorylated by ERK, restores telomere repeats through transcriptional activation of the telomerase catalytic subunit gene (hTERT), which contributes to senescence evasion [10]. The MAPK/ERK signaling pathway promotes survival by inhibiting the activation of pro-apoptotic BCL-2 family proteins such as BAX and BIM and inducing the expression of anti-apoptotic members of the BCL-2 family, such as BCL-2, BCL-XL, and MCL-1 [11]. This pathway also upregulates the expression of EMT-related genes, such as those encoding mesenchymal proteins and transcription repressors of epithelial genes, contributing to the induction and maintenance of the mesenchymal state in tumor cells [12]. The signaling pathway also activates cell motility and the invasiveness of cancer cells via the Rho/Rac-actin pathway and the upregulation of matrix metalloproteinase [13]. Furthermore, B-RAF induces angiogenesis through the involvement of HIF-1α and VEGF, and C-RAF (RAF-1) promotes endothelial cell survival, which plays a key role in the interaction between cancer and stromal cells [12].

### 2.2. The Role of the MAPK/ERK Signaling Pathway in HCC

The MAPK/ERK signaling pathway plays a pivotal role in tumorigenesis, and mutations in its components are highly prevalent in human cancers [14]. Approximately 20–30% of all human tumors are described as having mutations in one of the genes associated with RAS [9]. B-RAF mutations occur in ~50% of melanoma patients, and agents targeting the B-RAF showed improved clinical outcomes in these patients [15]. Additionally, inhibitors of MEK showed a significant effect on melanoma patients [15].

**Figure 2:**
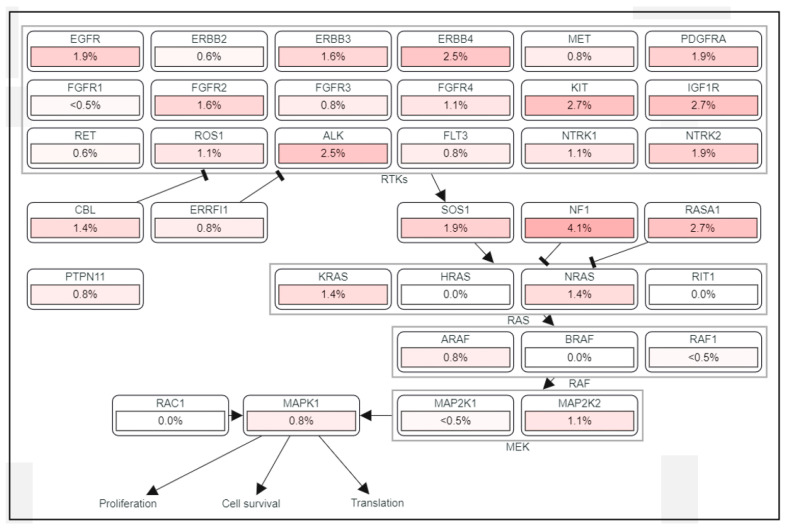
The frequency of mutations in the MAPK/ERK signaling pathway in HCC.

However, the importance of the MAPK/ERK pathway in human HCC has been neglected for a long time, mainly because mutations in RAS and RAF have not been frequently detected, found in less than 5% of HCC cases [16]. Despite a low frequency of mutations in the components of the MAPK/ERK signaling pathway (Figure 2), frequent activation of the signaling has been found in HCC patients [17]. MAPK/ERK signaling has prognostic significance as elevated expression levels of RAS effectors are highly correlated with a poor survival rate in HCC patients [18]. RAF-1 overexpression is also considered an independent marker of early tumor recurrence and poor prognosis [7,18]. Based on MEK/ERK expression and phosphorylation, MAPK/ERK signaling is considered to be activated in approximately 50% of early-stage HCC patients and in almost all patients with advanced-stage HCC [12]. It was demonstrated that the mRNA of RAF, MEK, and ERK was overexpressed in 33, 40, and 50% of HCC patients, respectively [19], and that MEK1/2 phosphorylation was increased seven-fold in HCC tissues compared to adjacent benign tissues [4].

Frequent activation of the MAPK/ERK signaling pathway in HCC is explained not so much by RAS and RAF mutations as by alternative mechanisms such as aberrant activation of upstream growth factors and receptors, downregulation of negative regulators of the MAPK/ERK signaling pathway, or upregulation of its positive modulators. In line with this, in most human HCCs, activation of the MAPK/ERK signaling pathway is observed in the presence of the wild-type genes of RAS, RAF, and downstream components [20].

### 2.3. Alternative Mechanisms Activating the MAPK/ERK Signaling Pathway in HCC

The activity of the MAPK/ERK signaling pathway is tightly regulated through the core pathway components, such as RTK, RAS, RAF, MEK, and ERK. Aberrant activation of the signaling pathway in human cancers is achieved via a constitutively activating mutation in genes encoding the core effector molecules as well as overexpression of the proteins. In addition to direct activation of the signaling pathway through the major effector molecules, MAPK/ERK signaling can also be affected by various modulators that activate and repress this signaling [5,21]. For example, activation of the MAPK/ERK signaling pathway can be achieved indirectly via either the suppression of regulators antagonizing the RAS-RAF-MEK-ERK cascade or the activation of modulators enhancing the signaling pathway (Table 1). 

The best examples of MAPK/ERK signaling modulators are RAS-GTPase activating proteins (RAS-GAPs) and RAS-guanine nucleotide exchange factor proteins (RAS-GEFs) [22,23] (Figure 1). Normal RAS proteins are maintained in equilibrium between a GTP-bound active state (RAS-GTP) and a GDP-bound inactive state (RAS-GDP). Although RAS proteins possess intrinsic catalytic function to hydrolyze GTP to GDP and GDP/GTP exchange activities, they require additional regulatory factors that facilitate and tightly regulate the GTP/GDP cycling process. RAS-GAPs stimulate the hydrolysis of bound GTP to GDP, allowing active RAS to switch to an inactive one, while RAS-GEFs play the opposite role in RAS-GTP/GDP cycling by stimulating the substitution of GTP for GDP in RAS [24]. Members of the RAS-GAP family, such as DAB2IP and RASAL1, are constitutively downregulated in several human HCC cell lines, including Hep 3B, SK-Hep-1, and PLC/PRF/5 [25]. Downregulation of at least one RAS-GAP was found in each sample in a large series of 88 human HCCs carrying wild-type *RAS* genes [20]. Thus, it is suggested that inactivation of RAS-GAPs is a major pathway leading to the activation of the MAPK/ERK signaling in human HCC. Expression levels of RASAL1, DAB2IP, and NF1 were downregulated in 73.8, 76.1, and 12.5% of cases, respectively, which showed mainly promoter hypermethylation and loss of heterozygosity (LOH) [7,20].

RAPGEF2, RASGRF2, RASGRP1, RASGRP4, and SOS are the members of RAS-GEFs [24]. Of these, the RASGRP1was expressed at significantly higher levels in HCC compared with tumor-surrounding tissues, and its high expression was remarkably associated with tumor size and stage [26]. Moreover, multivariate analysis revealed that the expression of RASGRP1 was an independent risk factor for HCC progression [26]. In addition, SOS was identified as a key molecule involved in diverse pathways activated in HCC, signifying its tumorigenic role in the cancer [27]. 

Sprouty proteins are another type of modulator that negatively regulate the MAPK/ERK signaling pathway by disrupting the GRB2/SOS complex and thus preventing RAS from switching to an active state [28]. Sprouty can also inhibit the signaling pathway at multiple levels. For example, both Sprouty and Sprouty-related proteins with EVH1 domains (SPRED) interfere with the phosphorylation of RAF [29]. Sprouty homologues are differentially expressed in cancer. Of these, Sprouty2 (SPRY2) and Sprouty4 (SPRY4) are downregulated compared to the corresponding non-tumor tissues, suggesting that they act as tumor suppressors in cancer via suppressing the MAPK/ERK signaling pathway [30]. Downregulation of SPRY2 is more prominent in HCC with poor prognosis, which is considered to confer an advantage to cancer cells with unrestricted ERK activity [31]. Overexpression of SPRED inhibits HCC cell proliferation in vitro and in vivo, by decreasing phosphorylation of ERK [32]. Decreased levels of SPRED expression correlate with characteristic features of tumor invasion and metastasis [32]. In addition, it was reported that the expression of SPRED-1 and SPRED-2 was reduced in 68% of tumor tissues compared to surrounding liver tissues [32]. 

RAF kinase inhibitor protein (RKIP) is also an endogenous inhibitor of the MAPK/ERK signaling pathway via the suppression of MEK activation and phosphorylation by RAF. RKIP dissociates a RAF-MEK complex and behaves as a competitive inhibitor of MEK phosphorylation [33]. Thus, loss of RKIP results in abnormal activation of the MAPK/ERK pathway, and reduced RKIP expression has been observed in a variety of human cancers [7,34]. RKIP is frequently downregulated in human HCC tissues when compared to non-tumor tissues and its downregulation contributes to the activation of the MAPK/ERK signaling pathway in HCC [35]. Dual-specificity phosphatase 1 (DUSP1) acts as a negative regulator of ERK [36]. Numerous members of the DUSP family are induced transcriptionally as a direct result of ERK activation, forming a negative regulatory loop [36]. A correlation was observed between decreased DUSP1 and increased ERK protein levels in HCC. Low expression of DUSP1 has been significantly correlated with increased tumor aggressiveness and reduced patient survival [37].

Various growth factors are also significant contributors to the activation of the MAPK/ERK signaling pathway in HCC in autocrine and paracrine manners. Epidermal growth factor (EGF) and transforming growth factor alpha (TGF-α) are the ligands that bind to EGFR and thus trigger subsequently the MAPK/ERK signaling pathway downstream of the RTK [38]. Increased expression of EGF and TGF-α occurs frequently in human HCC, especially at the early stages of human hepatocarcinogenesis, possibly signifying the role of the MAPK/ERK in the neoplastic transformation of hepatocytes [39,40]. Hepatocyte growth factor (HGF) is a paracrine cellular mitogenic factor. It is secreted by mesenchymal cells and targets primarily epithelial cells. In the liver, HGF is expressed and released by non-parenchymal hepatic stellate cells (HSC) and binds to HGFR (c-Met) on the surfaces of hepatocytes, triggering the MAPK/ERK signaling cascades and promoting the proliferation of the parenchymal cells [41,42]. HGF and HGFR are expressed at significantly high levels in HCC [43,44,45]. Binding of fibroblast growth factor (FGF) to fibroblast growth factor receptor also triggers the intracellular MAPK/ERK signaling pathway [46]. There are 22 types of FGFs and four FGFRs in humans. FGFRs contain immunoglobulin-like extracellular ligand-binding domains, an extracellular heparan sulfate (HS)-binding domain, a single transmembrane domain, and cytoplasmic tyrosine kinase domain [47]. Various FGFs, notably FGF1, FGF2, FGF8, FGF17, and FGF19, are elevated in HCC and stimulate the proliferation and invasion of HCC cells in an autocrine manner [48]. Likewise, at least one of the four FGFRs is upregulated in HCC [49]. The insulin-like growth factor (IGF) system regulates cellular metabolism, proliferation, differentiation, and apoptosis in the liver [50,51]. In the IGF system, there are two ligands, IGF-1 and IGF-2, and two receptors, insulin-like growth factor 1 receptor (IGF1R) and insulin-like growth factor 2 receptor (IGF2R). In addition, there is a family of seven high-affinity IGF-binding proteins (IGFBP1 to IGFBP7), which bind and stabilize IGF and regulate its accessibility to its receptors [52]. Signaling through IGF1R plays an important role in tumorigenesis in the liver and is one of the hallmarks of HCC [53,54]. Over-activation of IGF signaling can be induced by increased levels of IGF1R or excessive IGF, which subsequently leads to prolonged activation of the MAPK/ERK signaling pathway [55]. 

Non-coding RNAs, such as micro RNAs (miRNAs) and long non-coding RNAs (lncRNAs), regulate the MAPK/ERK signaling pathway. Numerous miRNAs have been found to regulate the MAPK/ERK signaling in HCC. For example, miR-4510 and miR-30a reduce the activity of the MAPK/ERK signaling pathway in HCC via directly suppressing RAS and RAF, respectively, while miR-330-5p and miR-487 exert the opposite effect on the activity by targeting Sprouty and SPRED, respectively which are negative regulators of the MAPK/ERK signaling pathway (see above) [56,57,58,59]. Downregulation of miR-4510 and miR-30a and overexpression of miR-330-5p and miR-487 have been reported in HCC [58,60]. Overexpression of a lncRNA, BRAF-activated non-coding RNA (BANCR) leads to activation of the MAPK/ERK signaling pathway in various cancers, including HCC [61,62,63,64]. Of note, downregulation of BANCR via shRNA-mediated knockdown inactivated the MAPK/ERK signaling in HCC cells, leading to the suppression of cellular proliferation and migration [65]. Other lncRNAs, such as IGF2AS, LL22NC03-N14H11.1, and URHC, are upregulated in HCC and known to promote tumorigenesis in the liver by activating the MAPK/ERK signaling [66,67,68]. On the contrary, lncRNAs RUNX1-IT1 and CASC2 inactivate the MAPK/ERK signaling pathway and their expression is significantly downregulated in HCC [69,70].

Exosomes are membranous vesicles released by cells, and they transport various kinds of intracellular biomolecules, including proteins, DNAs, messenger RNAs, miRNAs, and lncRNAs [71]. Exosomes are now recognized as a crucial player in intercellular communication both under normal and pathological conditions. Exosomes mediate communication between neoplastic cells and the tumor microenvironment, facilitating tumor growth, invasion, and metastasis [72]. In addition, intercellular communication among tumor cells can be achieved via exosomes, which contain various kinds of signal molecules. Exosomes released by cancer cells can activate the MAPK/ERK signaling in target cells by delivering growth factors, growth factor receptors, miRNAs, etc., to recipient cells [73,74]. Several studies have shown that HCC-derived exosomes trigger the MAPK/ERK signaling pathway in recipient cells [75].

Infection of HBV or HCV is known a major risk factor for HCC. Several molecular mechanisms have been proposed to explain the increased risk of HCC due to HBV/HCV infection, including genomic integration of viral DNA and inactivation of the apoptotic pathway [76,77]. Intriguingly, reports have shown that HBV can activate the MAPK/ERK pathway [78]. The HBV core antigen protein (HBcAg) has been shown to induce the production of IL-6 through activation of the MAPK/ERK signaling pathway in hepatocytes, which was blocked by chemicals antagonizing the MAPK/ERK signaling pathway [79]. In addition, studies have reported that the HCV core protein significantly activated the MAPK/ERK cascade [80]. Of note, it was reported that the activation of the MAPK/ERK signaling by the HCV core protein was blocked in the presence of an MEK1-specific inhibitor, suggesting that the viral protein may function at MEK1 or molecules upstream of MEK1 [81]. 

### 2.4. Animal Models for HCC with MAPK/ERK Activation

Numerous studies have identified that MAPK/ERK signaling is a major molecular pathway to hepatic tumorigenesis, not only in humans but also in experimental animal models [82]. To investigate hepatic carcinogenesis induced by the MAPK/ERK signaling pathway, many researchers have used a transgenic approach. Various transgenic mouse models have been developed using this method, which show high similarity to the pathological and molecular properties of human HCC [82,83]. A selection of genetically engineered mouse (GEM) models is summarized in Table 2, with emphasis on the activation of MAPK/ERK signaling.

Transgenic mice overexpressing c-Myc alone develop liver tumors inefficiently, with a long latency and low incidence [84]. However, the simultaneous expression of c-Myc and EGF significantly shortens the latency of HCC and causes liver cancer in 100% of mice [83,85]. Genetically engineered mouse (GEM) models expressing an activated β-catenin showed hepatomegaly or HCC with a long latency [86], but co-expression of activated RAS and activated β-catenin led to HCC in as little as 8 weeks [87]. Therefore, oncogenic cooperation with additional oncogenes is important in the development of HCC driven by MAPK/ERK signaling. Although GEM models for cancer are invaluable tools in deciphering roles of individual genes and cross-talk between molecular signaling pathways in human carcinogenesis, the development of GEM models via traditional transgenic and knockout techniques involves time-consuming and resource-demanding processes. 

A simple liver-specific transgenic approach using the hydrodynamic-based transfection (HT) method with the Sleeping Beauty (SB) transposase system has significantly improved the understanding of MAPK/ERK signaling in HCC [83,104]. Studies using this method have also shown that the combination of activated oncogenes or inactivated tumor suppressor genes with MAPK/ERK signaling can effectively induce HCC. Mice expressing activated AKT developed HCC approximately 6 months after HT [105], and co-expression of AKT with NRASG12V significantly shortened the latency [99]. Individual expression of cyclinD1 and c-Met did not induce tumors, while co-expression of the two caused HCC [95]. Likewise, neither Bmi1 nor RAS was sufficient to develop HCC; however, co-expression of Bmi1 and RAS induced HCC in 78.6% of mice within 15 to 30 weeks after HT [100]. Increased activities of Met and β-catenin are frequently present in human HCC [106]. Their co-expression in murine livers induced HCC as early as 7 weeks after HT, which is much shorter than that of tumor induction driven by individual expression [96].

The activation of MAPK/ERK signaling in HCC is induced not only by mutations in the core molecules of this signaling pathway, but also by various modulators (Section 2.3). The roles of these modulators in HCC development have been investigated in HT-based murine models. Lee et al. reported the development of HCC in transgenic mice models expressing the activated form of β-catenin together with a dominant-negative Sprouty2 (SPRY2Y55F) [101]. Similarly, although Sprouty2 downregulation alone did not induce any abnormality in the liver, cooperation with c-Met overexpression promoted HCC development in mice [31]. In addition, inactivation of Sprouty2 exerted a synergistic tumorigenic effect when combined with AKT activation, leading to rapid hepatocarcinogenesis through the MAPK/ERK signaling pathway [102].

Recent advances in gene editing make it easy to manipulate DNA using CRISPR (clustered regularly interspaced short palindromic repeats)/CAS9 (CRISPR-associated protein 9) technology [107]. Song et al. reported that NF1 knockdown in p53^−/−^Myc^+^Cas9 hepatocytes leads to the formation of tumors with constitutive activation of MAPK/ERK signaling [103]. In addition, inhibition by sorafenib or MEK inhibitors, AZD6244, and trametinib reduced tumor formation in NF1 knockout cells. Interestingly, tumors induced by the inactivation of NF1 showed overexpression of liver cancer stem cell markers such as HMGA2 and SOX9. This suggests that the activation of MAPK/ERK signaling could potentially have carcinogenic properties through the reprogramming of hepatocytes and activation of stem cell characteristics [103,108].

### 2.5. MAPK/ERK Signaling Pathway as a Therapeutic Target in HCC

There has been increasing interest in suppressing oncogenic signaling pathways via molecular-targeted therapies [21]. In particular, RTK has gained attention from both basic researchers and clinicians as a therapeutic target for the treatment of HCC [109]. Most tyrosine kinase inhibitors (TKIs) competitively inhibit ATP binding at the catalytic domains of diverse oncogenic TKs [110]. In addition to the ATP-competitive TKIs, which are called type I inhibitors, there are also non-ATP competitor TKIs, called type II and type III inhibitors. These inhibitors induce a conformational change in the target enzyme, thereby inactivating the kinase activity [109,111].

In HCC patients, sorafenib is the first clinically approved drug which targets RAF as well as RTKs such as VEGFR2, VEGFR3, PDGFR-β, and KIT [109]. Sunitinib, a multi-kinase inhibitor targeting PDGFR, VEGFR, KIT, and fms-like tyrosine kinase 3 (FLT-3), yielded unsatisfactory results compared with sorafenib in patients with advanced HCC. In a study, the median overall survival (OS) was 10.2 months for patients in the sorafenib group and 7.9 months for those in the Sunitinib group [112,113]. Erlotinib is a potent inhibitor of EGFR, and also showed anti-tumor activity in a phase II study of 38 patients with unresectable or metastatic HCC [4,114]. In a phase III study, HCC patients who received a combination of sorafenib and erlotinib showed a median OS of 9.5 months while those who received sorafenib plus placebo had a median OS of 8.5 months (Table 3). Lenvatinib has proven to be superior to sorafenib for HCC. Lenvatinib increased the overall survival (OS) in HCC patients who were not eligible for surgical removal of the tumor. Lenvatinib targets VEGFR, PDGFR, FGFR, and KIT and reduces angiogenesis and lympho-angiogenesis [115]. The problem of acquired resistance to lenvatinib has led to combination therapy such as with golvatinib [116]. 

Regorafenib has similar targets and structures as those of sorafenib and effectively represses STAT3 signaling through the activation of src homology 2 domain-containing phosphatase 1 (SHP1) [117,118]. It also suppresses V600-mutated B-RAF [117]. In a phase III study, sorafenib-refractory patients received either regorafenib as a second-line therapy or a placebo. Treatment with regorafenib led to a better OS compared with placebo treatment (median OS of 10.6 months vs. 7.8 months) [119]. Cabozantinib, an orally bioavailable multi-kinase inhibitor targeting VEGFR, MET, RET, and AXL, was approved for HCC patients [120]. The molecular mechanism of action of cabozantinib, a dual VEGFR/MET blockade, is noteworthy as the MET pathway is often activated after targeting VEGFR [120]. A phase III study evaluated the effect of Cabozantinib compared with a placebo on OS in patients with advanced HCC who had received prior sorafenib. The median OS of patients treated with cabozantinib was 10.2 months, compared to 8 months with the placebo, and the progression-free survival (PFS) was 5.5 months, compared to 1.9 months with the placebo [109,121]. Tivantinib, a selective oral MET inhibitor, improved OS and PFS compared to a placebo in a phase II study of patients with high MET-expressing HCC who were previously treated with sorafenib. However, in a phase III study investigating the effectiveness of treating patients with MET-high hepatocellular carcinoma previously treated with a systemic therapy, tivantinib did not improve OS when compared with a placebo [122]. 

Ramucirumab, a monoclonal antibody against VEGFR2, showed an improvement in the median OS for patients with alpha-fetoprotein (AFP) >400 ng/mL who had been previously treated with sorafenib [123]. In the REACH-2 trial, a phase III study in patients previously treated with sorafenib, the median OS was 8.5 months for patients receiving ramucirumab and 7.3 months for those receiving a placebo [123,124]. It is intriguing that the serum AFP level in patients is an important prognostic factor and a predictive biomarker for ramucirumab survival benefit [125,126].

To date, MAPK/ERK signaling-based target therapy for HCC has focused on the inhibition of RTKs (Table 3) [109]. As a new therapeutic approach, the immune checkpoint is emerging as an attractive target for the treatment of HCC. The rationale for targeting the immune checkpoint is based on the observation that the expression of programmed death-ligand 1 (PD-L1) in cancer cells can evade the immune system through its interaction with programmed death-1 (PD-1) located on the surfaces of activated T cells, B cells, natural killer cells, and myeloid cells [127]. Nivolumab, a fully human monoclonal antibody binding to PD-1, inhibits the interaction of PD-1 with PD-L and suppresses the immune checkpoint signaling. The phase III CheckMate 459 study compared the clinical efficacy and safety of nivolumab with sorafenib as first-line therapy in patients with advanced HCC [128]. Although the study showed that nivolumab treatment led to clinically meaningful improvements in OS compared with sorafenib (16.4 versus 14.7 months), the result was not statistically significant [128]. Another phase III study, KEYNOTE-240, investigated the efficacy and safety of pembrolizumab, a humanized monoclonal antibody blocking PD-1, as a second-line therapy in patients with advanced HCC who had been previously treated with sorafenib [129]. This study also failed to show statistically significant improvements in OS and PFS in the pembrolizumab group, compared with the placebo group. Although targeting the immune checkpoint alone showed somewhat discouraging clinical outcomes, the simultaneous inhibition of the immune checkpoint and VEGF revealed unprecedented clinical efficacy in HCC patients. In a global, open-label, phase III trial, patients with unresectable HCC who had not been previously treated with systemic therapy were randomly assigned to receive either atezolizumab (targeting PD-L1) plus bevacizumab (targeting VEGF-A) or sorafenib. Treatment with atezolizumab combined with bevacizumab resulted in better OS and PFS outcomes compared with sorafenib treatment [130,131]. Moreover, a recent updated analysis showed a median OS of 19.2 months for atezolizumab plus bevacizumab and 13.4 months for sorafenib at 12 months of additional follow-up from the primary analysis (Table 3) [132]. These results provide the longest survival to date for a phase III study in advanced HCC, supporting the continued safety and efficacy of atezolizumab plus bevacizumab. The combinational therapy has become the new standard of care for most patients with advanced HCC [131,133].

### 2.6. Therapeutic Interventions of the MAPK/ERK Signaling Pathway in Other Cancers

As described in Section 2.5, MAPK/ERK signaling-based target therapy in HCC has been limited to the inhibition of RTKs and RAF. In contrast, therapeutic interventions of the MAPK/ERK signaling at different levels have been clinically attempted in other cancers, and so reviewing the clinical studies might bring new insights into MAPK/ERK signaling-based therapy for HCC.

Due to frequent observations of a constitutively activating mutation in *RAS* in human cancer, various attempts have been made to directly inhibit the RAS proteins. However, RAS is difficult to treat with drugs due to the lack of suitable docking sites for small-molecule inhibitors with high affinity and selectivity [134]. A few exceptions involve targeting oncogenic RAS containing a specific activating mutation. Small-molecule cysteine-reactive inhibitors that covalently modify the mutant KRASG12C protein, such as AMG-510 and MRTX849, have been clinically tested for patients with non-small cell lung cancer harboring the KRASG12C mutation and showed promising efficacies [135,136]. Because of the extreme challenges in developing an inhibitor directly targeting RAS proteins, in general, indirect approaches have been adopted to modulate the functions of RAS. Farnesylation, which transfers the 15-carbon farnesyl moiety to the C-terminal cysteine of RAS proteins, is pivotal in the proper membrane localization and physiological function of the proteins. Thus, targeting the farnesylation pathway has attracted significant attention as a therapeutic strategy to inhibit RAS and subsequently suppress the MAPK/ERK signaling cascades [137,138]. However, farnesyltransferase inhibitors such as lonafarnib and tipifarnib have shown dismal clinical results in patients with RAS-driven cancers due to the restored membrane association of RAS proteins due to the compensatory function of geranylgeranyl transferase I [139].

Considering that RAS proteins are almost impossible to treat with drugs, molecules downstream of RAS are more attractive targets to suppress activated MAPK/ERK signaling in cancers. Selective B-RAF inhibitors such as vemurafenib and dabrafenib have been approved for metastatic and unresectable B-RAF-mutated melanomas [15]. These B-RAF inhibitors achieved desirable clinical efficacy for melanoma patients with mutant B-RAF and significantly improved the OS and PFS of the patients [15]. Since MEK is rarely mutated in human cancer, it was not considered an effective target in the past, but with increasing knowledge of the role of the MAPK/ERK signaling in cancer, targeting MEK is attracting more and more attention [140]. Among the MEK inhibitors, selumetinib, recently approved by the FDA, was effective in patients with relapsed low-grade serous ovarian cancer (LGSOC) in a phase II clinical trial and patients with neurofibromatosis type 1 [141,142,143]. Another MEK inhibitor, trametinib, showed an improvement in PFS compared with the current standard of care in patients with recurrent LGSOC [144]. In addition, in patients with histiocytic neoplasms who were treated with cobimetinib, an orally administered MEK inhibitor, the overall response rate was remarkably 89% [145].

Although there are currently no FDA-approved inhibitors targeting ERK, the recognition that ERK reactivation arises in resistant cancers following prolonged treatment with RAF and MEK inhibitors has generated renewed interest in targeting ERK (see Section 2.7). Selective ERK1/2 inhibitors, including BVD-523, LY3214996, and ASTX-029, are currently under preclinical and clinical evaluation for various solid cancers with RAS or RAF mutations [33,139,146,147,148]. In particular, BVD-523 showed anti-proliferative activity in cell lines of colorectal and pancreatic cancers with B-RAF and K-RAS mutations, respectively [149,150]. Unfortunately, despite these continuous efforts to target the MAPK/ERK signaling pathway in cancers, cancer cells ultimately develop resistance to given MAPK/ERK inhibitors. Relapse of cancer acquiring resistance remains a major challenge to overcome in the MAPK/ERK signaling-based target therapy for cancer.

### 2.7. Resistance of Cancer Cells to Drugs Targeting the MAPK/ERK Signaling Pathway

Although targeted therapies against RTKs have high potency and selectivity, they are susceptible to the emergence of drug resistance [151]. Reactivation of MAPK/ERK signaling in cancer can be achieved through alterations or mutations in downstream RAS, RAF, MEK, or ERK [152]. Resistance to RTK inhibitors can also arise through the acquisition of a resistance mutation within the RTK, minimizing the activity of the inhibitors. The emergence of the EGFRT790M mutation has been frequently observed following EGFR-targeted therapy in non-small cell lung cancer [153].

Despite the initial clinical benefits of B-RAF inhibitors, continuous treatment with B-RAF inhibitors results in drug resistance. For example, all ATP-competitive B-RAF inhibitors, including vemurafenib, dabrafenib, and sorafenib, gave rise to reactivation of the MAPK/ERK signaling by triggering conformational changes in RAF molecules or by acquiring an activating mutation in the downstream MEK and ERK [15,154]. Recently, to overcome the drug resistance of the first-generation inhibitors, second-generation RAF inhibitors such as PLX8394, BGB283, TAK-580, and LXH254 have been developed and are currently undergoing clinical evaluations in advanced solid tumors [140,152,155]. In addition, dual inhibition of RAF and MEK or RAF and ERK has been clinically tested for reduced cancer relapses [15]. Cancers treated with MEK inhibitors also develop resistance to the drugs via reactivation of the MAPK/ERK pathway. Several mechanisms have been proposed for the reactivation of the signaling in cancer following treatment with MEK inhibitors. Reactivation can occur through alterations or mutations to upstream molecules such as RTKs, RAS, RAF, or NF1, reinforcing the signaling pathway [141,156]. Resistance to MEK inhibitors can also occur when a mutation has arisen in MEK, leading to impaired drug binding to MEK [141].

Apart from reactivation of the MAPK/ERK signaling pathway, cancers acquire resistance to MAPK/ERK signaling-based target therapy by recruiting an alternative signaling pathway that induces cell proliferation and growth. In particular, activation of the PI3K/AKT and yes-associated protein (YAP) signaling pathways stand out as the bypass mechanisms for resistance to therapeutic interventions of the MAPK/ERK signaling [141]. For example, colorectal cancer cells with an activating mutation in K-RAS became resistant to the inhibition of EGFR and MEK via activation of PI3K/AKT [157]. In a similar fashion, melanoma showed resistance to the inhibition of MEK via the upregulation of AKT and YAP, which provided cancer cells with enhanced survival and oncogenic activity [158]. Moreover, human non-small cell lung carcinomas with adaptive resistance to MEK inhibitors were highly susceptible to treatment with an additional YAP inhibitor, which suppresses the emergence of resistant cancer cells [159].

Chromatin remodeling or epigenetic mechanisms are also significantly involved in drug resistance to MAPK/ERK inhibition. Melanoma cells with the BRAFV600E mutation became resistant to RAF inhibition (dabrafenib) alone or in combination with MEK inhibition (trametinib) through downregulation of histone deacetylase SIRT6 [160]. Interestingly, only the haploinsufficiency of SIRT6 was able to confer resistance to RAF and/or MEK inhibition by increasing H3K56 acetylation at the IGFBP2 locus, leading to the activation of the IGF and its downstream signaling [160]. Genetic and epigenetic changes in cancers contribute significantly to drug resistance and sensitivity. Understanding the resistance mechanisms will lead to an effective MAPK/ERK signaling-targeted therapy for various human cancers.

## 3. Perspectives and Conclusions

A precise understanding of the molecular pathway toward carcinogenesis will allow the patient response to targeted therapies to be predicted, which can have a substantial impact on clinical decision-making. The development of HCC is a complex, multi-step process accompanied by alterations in multiple signaling cascades [4]. Among the various oncogenic signals, MAPK/ERK signaling is activated in approximately 50% of early-stage HCC patients and in the majority of patients with advanced HCC [14]. Numerous studies have shown that MAPK/ERK signaling plays a decisive and central role in the development of HCC [17]. Although upregulation of the MAPK/ERK signaling pathway is frequently found in HCC, mutations in intracellular effectors of this signaling are infrequent in patients with HCC. This suggests that an alternative mechanism should be present to activate the signaling pathway in HCC. To date, RTKs, the upstream components of the MAK/ERK signaling pathway, are the most favored molecular targets for the treatment of HCC. With less than desirable clinical outcomes from RTK inhibitors alone, as exemplified by sorafenib, combinational therapy with other types of target therapy has been considered for the treatment of HCC. 

To date, MAPK/ERK signaling-based target therapy in cancer has focused on the core molecules in the signaling (see Section 2.6). There are many different types of positive modulators of the signaling pathway (Table 1). Inhibition of novel targets that play critical roles in the activation of the MAPK/ERK signaling pathway should be considered. We look forward to seeing how effective the novel target therapies will be in HCC under preclinical settings using transgenic animal models with activated MAPK/ERK signaling as well as in clinical trials.

## Figures and Tables

**Table 1:** Effects of various modulators on the MAPK/ERK signaling pathway in HCC.

Modulators	Examples	Effect
RAS-GAP	DAB2IP, RASAL1, RASAL2, NF1	Suppression
RAS-GEF	RAPGEF2, RASGRF2, RASGRP1, RASGRP4, SOS	Activation
Sprouty	SPRY2, SPRY4, SPRED1, SPRED2	Suppression
RKIP and DUSP	RKIP, DUSP1	Suppression
Growth factor	EGF, TGF-α, HGF, FGF, IGF	Activation
MicroRNA	miR-330-5p, miR-487/miR-4510, miR-30a	Activation/Suppression
Long non-coding RNA	BANCR, IGF2AS, URHC LL22NC03-N14H11.1/RUNX1-IT1 and CASC2	Activation/Suppression
Exosome	mRNAs, Proteins, miRNAs	Activation/Suppression
Hepatitis virus	HBV, HCV	Activation

**Table 2:** Genetically engineered mouse models for HCC development.

Modulation System	Genes	Latency (Weeks)	Characteristics	Refs.
Traditional transgenic and knockout techniques	EGF	30	HCC	[40,85]
	EGF + c-Myc	12	HCC	[83,85]
	HGF	85	HCC	[88,89]
	HRAS^G12V^ + β-catenin^Δex3^	8	well-differentiated HCC	[87]
	KRAS^G12D^	50	well-differentiated HCC	[90]
	KRAS^G12D^ + HBx	40	poorly differentiated HCC	[90]
	MET	30	HCC	[91,92]
	PDGFR	60	well-differentiated HCC	[93]
HT and Sleeping Beauty transposon	c-Met + Spry2^Y55F^; Arf^−/−^	14	HCC	[31]
	HBx + shp53	20	HCC	[94]
	hMet + ΔN90-β-catenin	7	HCC	[91,95,96]
	hMet + CCND1	25	HCC	[95]
	HRAS^G12V^ + cMyc	6	moderately differentiated HCC	[83,97]
	HRAS^G12V^ + shp53	4	poorly differentiated HCC	[83,97]
	NRAS; Arf^−/−^	40	mixed HCC and ICC	[98]
	NRAS^G12V^ + myr-AKT	4	mixed HCC and ICC	[99]
	RAS^V12^ + Bmi1	15	HCC	[100]
	NRAS^G12V^ + ΔN90-β-catenin	13	HCC	[101]
	Spry2^Y55F^ + ΔN90-β-catenin	24	HCC	[101]
	Spry2^Y55F^ + myr-AKT	8	HCC with emperipolesis	[102]
HT and CRISPR/Cas9	Myc + Cas9; sgNf1; p53^–/–^	5	HCC	[103]

Abbreviations: HT, hydrodynamics-based transfection; ICC, intrahepatic cholangiocarcinoma.

**Table 3:** Phase III clinical trials of molecular-targeted therapies for HCC.

Drug	Therapeutic Targets	NCT Number (Study Design)	Median OS (Months)	Median PFS (Months)
Sorafenib	VEGFR, PDGFR, RAF, KIT	NCT00105443 (Sorafenib vs. Placebo)	10.6 vs. 7.9	NR
Sunitinib	VEGFR, PDGFR, KIT, RET	NCT00699374 (Sunitinib vs. Sorafenib)	7.9 vs. 10.2	3.5 vs. 2.9
Erlotinib	EGFR	NCT00901901 (Erlotinib + Sorafenib vs. Placebo + Sorafenib)	9.5 vs. 8.5	NR
Lenvatinib	VEGFR, PDGFR, FGFR, RET, KIT	NCT01761266 (Lenvatinib vs. Sorafenib)	13.6 vs. 12.3	7.4 vs. 3.7
Regorafenib	VEGFR, PDGFR, FGFR, KIT, RET, B-RAF	NCT01774344 (Regorafenib vs. Placebo)	10.6 vs. 7.8	3.1 vs. 1.5
Cabozantinib	VEGFR, MET, AXL, RET	NCT01908426 (Cabozantinib vs. Placebo)	10.2 vs. 8.0	5.2 vs. 1.9
Tivantinib	HGFR	NCT01755767 (Tivantinib vs. Placebo)	8.4 vs. 9.1	2.1 vs. 2.0
Ramucirumab	VEGFR2	NCT02435433 (Ramucirumab vs. Placebo)	8.5 vs. 7.3	2.8 vs. 1.6
Nivolumab	PD-1	NCT02576509 (Nivolumab vs. Sorafenib)	16.4 vs. 14.7	3.7 vs. 3.8
Pembrolizumab	PD-1	NCT02702401 (Pembrolizumab vs. Placebo)	13.9 vs. 10.6	3.0 vs. 2.8
Atezolizumab and Bevacizumab	PD-L1 and VEGF-A	NCT03434379 (Atezolizumab + Bevacizumab vs. Sorafenib)	19.2 vs. 13.4	6.9 vs. 4.3

Data accessed on the ClinicalTrials.gov online database. Abbreviations: KIT, c-Kit proto-oncogene; RET, rearranged during transfection; OS, overall survival; PFS, progression-free survival; NR, not reported.

## Data Availability

Not applicable.
